# A clinical randomized trial on the use of atorvastatin in patients with sepsis or septic shock: effects on endothelial function

**DOI:** 10.1186/cc11999

**Published:** 2013-03-19

**Authors:** K Prado, C Ribeiro, T Furian, R Pinto Ribeiro, D Silvello, L Rohde, N Clausell, L Becker

**Affiliations:** 1 Hospital de Clinicas de Porto Alegre, Brazil

## Introduction

Sepsis and septic shock are complex inflammatory syndromes. Multiple cellular activation processes are involved, and many humoral cascades are triggered. Presumably, endothelial cells play a pivotal rule in the pathogenesis of sepsis, not only because they may influence the inflammatory cascade but also because, upon interaction with excessive amounts of inflammatory mediators, the function of these cells may become impaired. It is likely that a general dysfunction of the endothelium is a key event in the pathogenesis of sepsis [1]. HMG-CoA-reductase inhibitors have been shown to exhibit pronounced immunomodulatory effects independent of lipid lowering. Most of these beneficial effects of statins appear to involve restoring or improving endothelial function [2]. We hypothesize that statins can improve endothelial dysfunction in septic patients.

## Methods

A double-blinded, placebo-controlled, randomized trial was undertaken. We enrolled adult patients within 24 hours of severe sepsis or septic shock diagnosis and randomized them to placebo or atorvastatin 80 mg/day for a short term. Endothelial dysfunction was assessed measuring plasmatic levels of IL-6, ET-1, VCAM-1 by ELISA and measuring flow-mediated vasodilatation of the brachial artery at basal, 24 and 72 hours after randomization.

## Results

We studied 47 patients, 24 in the placebo group (mean age 52 ± 20 years, 29.1% male; APACHE II risk score 23.5 ± 7.3) and 23 in the statin group (mean age 49.5 ± 18 years, 53.4% male; APACHE II risk score 23 ± 6.9). The baseline characteristics of the placebo group were similar to statin patients as well as the mean length of stay in the ICU (8.6 ± 7.4 and 9.1 ± 8 days, respectively) and the time on vasopressors (49.3 ± 47.1 and 59 ± 91.1 hours, respectively). No significant difference was observed on the temporal variation of biomarker levels (IL-6, VCAM-1, ET-1) between treatment and control groups. The intrahospital mortality rate was 26% in the statin group and 45% in the placebo group (*P *= 0.17).

## Conclusion

Our data showed no benefit with the use of a potent statin acutely in patients with sepsis or septic shock with regards to improvement in endothelial function.
